# Comparison of Metabolic and Pulmonary Variables Between Real-Life and Mixed Reality Pickleball

**DOI:** 10.3390/jfmk10030346

**Published:** 2025-09-11

**Authors:** Setareh Zarei, Matahn Blank, Jamaal Bovell, Dustin W. Davis, Jacob Baca, Michael W. H. Wong, Brett Abarbanel, James W. Navalta

**Affiliations:** 1 Department of Kinesiology and Nutrition Sciences, University of Nevada (Las Vegas), Las Vegas, NV 89154, USA; setareh.zarei@unlv.edu (S.Z.); blankm1@unlv.nevada.edu (M.B.); bovell@unlv.nevada.edu (J.B.); dustin.davis@unlv.edu (D.W.D.); bacaj1@unlv.nevada.edu (J.B.); michael.wong@unlv.edu (M.W.H.W.); 2 International Gaming Institute, University of Nevada (Las Vegas), Las Vegas, NV 89154, USA; brett.abarbanel@unlv.edu

**Keywords:** paddle sport, injury, paddle sports, wearable technology, headsets, Meta Quest

## Abstract

**Background:** Pickleball is one of the fastest growing sports, and the use of virtual reality is also fast growing. Because the physiological responses in real life (IRL) vs. virtual reality are unknown, the purpose of this research was to compare heart rate, metabolic and pulmonary measures IRL vs. mixed reality (MR) during pickleball activity. **Methods**: Eleven adult participants were outfitted with a portable metabolic unit, heart rate monitor, and virtual reality headsets. Participants played simulated pickleball for 5 min IRL and 5 min in MR. Dependent variables included average heart rate (HR [beats per minute (bpm)], ventilation (VE [L/min]), tidal volume (VT [L]), respiratory frequency (Rf [breaths per min]), respiratory exchange ratio (RER), percent of calories from fat (FAT%), percent of calories from carbohydrate (CHO%), energy expenditure (EE [kilocalorie (kcal]), and VO_2_ (mL/kg/min). Data were analyzed using paired *t*-tests with significance accepted at *p* < 0.05. Effect size measurements were determined by interpretation of small (*d* = 0.2), medium (*d* = 0.5), and large (*d* = 0.8). **Results**: All metabolic and pulmonary variables except for FAT% were higher during IRL when compared with MR with effect sizes ranging from median to large. **Conclusions**: The results of this study provide evidence that playing pickleball IRL results in greater physiological responses in comparison to MR. Since MR demands less exertion and substrate use than IRL this result can be beneficial for training purposes with the added potential of reduced injury.

## 1. Introduction

The development of high-tech wearable devices revolutionizes life by seamlessly integrating into daily routines and enhancing convenience, health monitoring, and connectivity. Virtual reality (VR) as one of the latest technologies on the market, provides a high sense of realism [1] by displacing a person to an imaginary location, physically blocking out the real world, and replacing it with a computer-generated world [2]. This is usually achieved using a head-mounted display, known as a cave automatic virtual environment system (an immersive VR environment where projectors are directed onto between three and six of the walls of a room-sized cube) [3].

Given these immersive qualities, VR has gained attention not only in the domains of entertainment and education but also physical activity. By replicating real-world environments and overlaying engaging stimuli, VR enables users to exercise in interactive, simulated settings such as gyms, parks, or sports arenas. This integration of auditory and visual stimulation creates opportunities to support physical activity in novel, engaging ways. For example, by simulating immersive exercise environments such as virtual cycling through scenic landscapes, which has been shown to enhance user motivation and energy expenditure [4,5].

Beyond educating and training users with specific skills [6], the primary aim of introducing VR technology to the general consumers, particularly within the realm of fitness, is to enhance user engagement and commitment [7]. Through the implementation of an exercise regimen that directs focus away from discomforting bodily sensations, such as muscle soreness and increased breathing, in addition to postponing the onset of boredom and fatigue [8,9], it is potentially feasible to encourage exercising at a higher intensity in each session. As a result, VR is gaining popularity as an alternative form of exercise [10] to help participants adopt behaviors and performances similar to those in real-world activities [11], contributing to the expected rapid expansion of VR with expected growth to reach 30.33 m units by 2030 [12].

Mixed reality (MR) is a form of immersive technology that blends virtual content with elements of the physical world, allowing users to interact with both simultaneously. Unlike standard virtual reality (VR), which fully immerses users in a digitally generated environment and often blocks out the real world, MR overlays digital stimuli onto the real environment by maintaining users’ awareness of physical space while enhancing it with interactive virtual elements. This distinction is particularly important in physically active settings, as MR enables real-world movement patterns such as stepping, pivoting, and reaching within a constrained but realistic space. As technological innovations continue to permeate leisure and fitness activities, questions arise about the physiological and perceptual effects of MR compared to traditional in-person (IRL) activity. In dynamic sports like pickleball, characterized by quick direction changes, reactive play, and complex motor demands, MR may uniquely support realistic training while moderating physical intensity. By allowing movement in a familiar physical environment enhanced with gamified virtual cues, MR could influence both energy expenditure and user engagement differently than fully immersive VR. Investigating these differences is critical to understanding how MR can be used not only for skill development but also for accessible, lower-impact training and rehabilitation in sport science.

Virtual and mixed reality platforms are uniquely positioned to replicate the intermittent, moderate-to-vigorous metabolic profile of pickleball while offering enhanced safety and controllability. By simulating rallies, quick directional changes, and sustained rallies with adjustable difficulty, VR/MR may elicit comparable cardiovascular and pulmonary responses to IRL play. Importantly, these environments may reduce external risk factors such as accidental collisions or ball impact, while still promoting aerobic and anaerobic engagement. This suggests that VR/MR may offer a viable training modality for populations seeking the metabolic benefits of pickleball without the full physiological or injury risks of in-person play.

Pickleball, a racquet sport, has become one of the fastest-growing sports in the United States, attracting significant research interest in recent years [13]. Since its inception in 1965, pickleball has increased in popularity with a substantial jump in player numbers from 4.8 million to 8.9 million between 2021 and 2022 [14,15]. As pickleball’s popularity has increased, so too has the incidence of injuries [16,17]. Beyond injury risk, it is also important to understand the physiological demands of pickleball to better contextualize its health and performance implications. From a physiological perspective, pickleball is generally classified as a moderate-intensity sport. In older adult participants, mean heart rate during match play averaged 108.8 ± 16.7 bpm (~50.9 ± 11.2% heart rate reserve) and elicited approximately 4.1 ± 1.0 METs (~52.5% VO_2_ reserve) [18]. Additionally, recreational singles and doubles pickleball players achieved mean heart rates of ~111 bpm (70–71% of predicted HRmax), with over 70% of active play time categorized as moderate to vigorous intensity [19]. The game also involves intermittent high-intensity actions such as dashing to the net, pivoting, and extending rallies that demand both aerobic and anaerobic energy systems. Therefore, given both the injury risk and the physiological demands of pickleball, it may be beneficial for people to participate in pickleball in an MR situation. This approach can provide training that potentially decreases injury IRL due to less physiological stress in the MR environment. Before this evaluation can be performed, a comparison of physiological measures while playing pickleball in MR and IRL must first be conducted.

Although immersive VR has been shown to elicit higher exercise intensity and VO_2_ engagement compared to matched screen-based workouts (e.g., VR boxing via FitXR™) while enhancing enjoyment and affect [20], other exergame research suggests VR may provoke lower heart rate responses than traditional exercise yet still improves user adherence and motivation [21]. However, the physiological implications of mixed reality in dynamic racquet sports remain unexplored, particularly in comparison with real-world play. This study fills this gap by directly comparing MR and in-real-life physiological responses during pickleball. To our knowledge, no study to date has examined the physiological effects of MR vs. IRL in an exercise setting. With the absence of such a study, this investigation aimed to bridge the gap in existing research by systematically comparing physiological measures in pickleball gameplay across real-world and mixed reality environments. Specifically, we measured heart rate, metabolic and pulmonary measures. It was hypothesized that heart rate, metabolic and pulmonary measures would be lower during MR since this environment seems likely to induce a reduction in physiological stress levels [5].

The findings of this study are expected to provide valuable insights into the effectiveness of MR as a tool for sports training and its potential to change traditional training paradigms.

## 2. Materials and Methods

### 2.1. Participants

Eligible participants were healthy adults aged 18 years or older who felt comfortable playing pickleball and participating in a virtual environment. Eleven adult participants (female *n =* 4, male *n =* 7, identified otherwise *n =* 0, age = 31.39 ± 8.84 years; height = 171.82 ± 6.87 cm; mass = 71.79 ± 12.42 kg) were recruited for this study after completing a written informed consent, followed by the American College of Sports Medicine health risk questionnaire [22] to determine study eligibility. The anthropometric information was self-reported. Individuals were excluded if they reported cardiovascular, metabolic, or renal disease; had musculoskeletal injuries that could prevent pickleball participation; were pregnant or suspected pregnancy; or if their health risk questionnaire responses indicated that medical clearance was necessary before engaging in moderate to vigorous exercise. Participants were recruited for this study via convenience sampling from the university community. This study was approved by the Institutional Review Board (IRB approval #UNLV-2024-318).

### 2.2. Protocol

In this study’s setup, all participants wore a Meta S3 VR headset (Meta Horizon, Menlo Park, CA, USA), a Polar H10 chest strap heart rate monitor (Polar Electro Inc., Kempele, Finland) to measure HR, and a COSMED K5 portable metabolic analysis system (Rome, Italy) to measure metabolic and pulmonary measures. The Polar H10 was worn around the chest, and the K5 was secured to participants’ backs with small sampling lines connected to a facemask. The outcome measures included HR, metabolic and pulmonary measures. Heart rate measures are average heart rate (HR [beats per minute (bpm)]). Metabolic measures were VO_2_ (mL/kg/min), percent of calories from fat (FAT%), percent of calories from carbohydrate (CHO%), energy expenditure (EE [kilocalorie (Kcal)]), and respiratory exchange ratio (RER). Pulmonary measures included ventilation (VE [L/min]), tidal volume (VT [L]), and respiratory frequency (Rf [breaths per min]). 

This study consisted of a single testing day of acute singles pickleball activity. A simulated match was completed against an investigator for IRL testing inside the UNLV Exercise Physiology Lab. Each participant played pickleball for a total of 10 min–5 min in each condition (MR and IRL), with a five-minute rest period in between the sessions.

**Figure 1:**
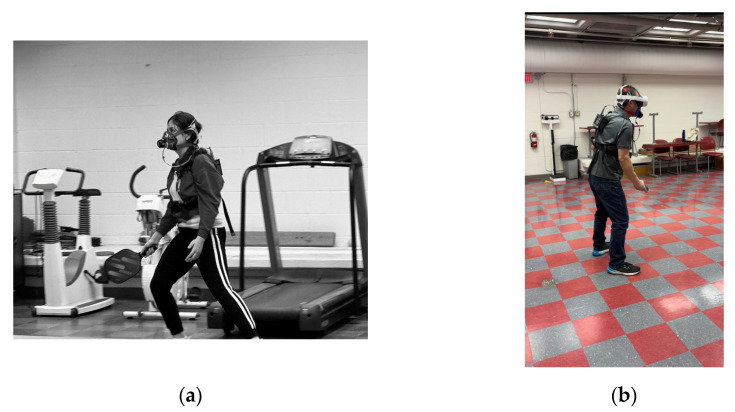
(**a**) Example of experimental device setup utilized in the investigation for IRL condition. (**b**) Example of experimental device setup utilized in the investigation for MR condition.

In the MR condition, each participant wore a COSMED K5, Polar H10 heart rate monitor and a headset, carrying two controllers in two hands (see Figure 1). Right hand participants used the right controller as the paddle and left-hand participants left controller. Pickleball One application was chosen for running the MR condition and two different accounts were used in each headset so players could join the game separately. The virtual environment was created using the multiplayer/mixed reality option. To use this option, the room in which testing occurred was scanned. The pickleball net was placed in the middle of the room, and boundaries were set for the position that the participants were standing in. 

In the IRL condition, participants wore a COSMED K5, Polar H10 heart rate monitor, and a headset, but carried one paddle this time (see Figure 1). The location was the same for both conditions.

In both conditions, participants used the same playing hand for each 5 min session (If a participant used their dominant hand for the first 5 min, they continued playing with the same hand for the remaining 5 min). The starting condition (MR or IRL) was counterbalanced. The number of participants starting IRL was equal to the number of participants starting in MR. A short warm up was performed prior to the VR session for familiarity purposes. Participants were assigned to starting conditions in an alternating order as they arrived (e.g., first participant began in the IRL condition, second in MR condition, third in IRL, etc.). This systematic approach ensured an approximately equal number of participants began in each condition, serving as a form of partial counterbalancing to control for order effects.

### 2.3. Data Analysis

Pickleball is typically not a steady-state activity, which can influence VO_2_ measurements. To account for this, VO_2_ values were averaged over the final two minutes of gameplay. Data from the mixed reality and IRL conditions were analyzed using a paired *t*-test with significance set at *p* < 0.05, conducted in SPSS Statistics (IBM SPSS Statistics, Version 28.0.1.0, Chicago, IL, USA). Before analysis, the assumption of normality for the difference scores was assessed using the Shapiro–Wilk test. The data were evaluated to ensure that they were measured on an interval or ratio scale and that the paired observations were independent from other participant pairs. Effect sizes were calculated using Cohen’s *d* and interpreted as small (*d* = 0.2), medium (*d* = 0.5), and large (*d* = 0.8) [23].

## 3. Results

**Figure 2:**
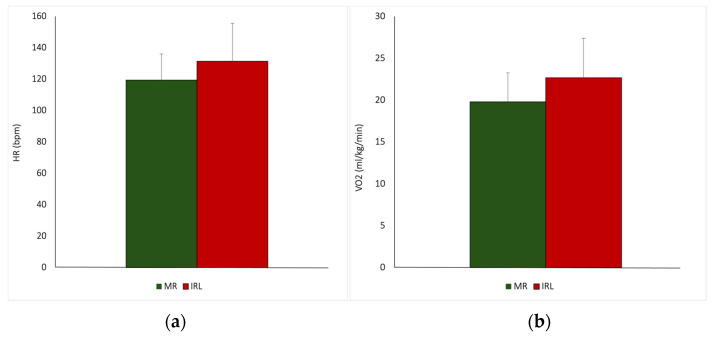
(**a**) Example of pulmonary measures presented in figure form. (**b**) Example of metabolic measures presented in figure form.

Significant differences between MR and IRL were observed for all the measurements except for FAT%. The heart rate, pulmonary, and metabolic data were as follows: (see Table 1 and Figure 2, and Table 2 and Figure 2).

- Heart rate and pulmonary measures.

**Table 1:** Pulmonary measures during pickleball gameplay in MR and RL.

Pulmonary Measures and HR	MR	IRL	*p*	*d*
HR (bpm)	119.38 ± 16.64	131.36 ± 24.21	0.002	1.12
Rf (breath per minute)	35.06 ± 5.28	37.85 ± 6.28	0.040	0.58
VT (L)	1.04 ± 0.17	1.15 ± 0.24	0.012	0.80
VE (L/min)	35.93 ± 6.95	43.05 ± 11.52	0.003	1.05

Heart rate (HR); respiratory frequency (Rf); VT tidal volume (VT); ventilation (VE).

- Metabolic Measures

**Table 2:** Metabolic measures during pickleball gameplay in MR and RL.

Metabolic Measures	MR	IRL	*p*	*d*
RER	0.80 ± 0.03	0.83 ± 0.03	0.01	0.84
FAT (%)	67.28 ± 11.76	55.66 ± 11.91	0.01	0.83
CHO (%)	32.72 ± 11.76	44.34 ± 11.91	0.01	0.84
EE (Kcal)	6.42 ± 1.53	7.26 ± 2.03	0.02	0.69
VO_2_ (mL/kg/min)	19.77 ± 3.47	22.68 ± 4.71	0.004	0.99

Respiratory exchange ratio (RER), percentage of calories from fat (FAT); percentage of calories from carbohydrate (CHO); energy expenditure (EE); volume of oxygen (VO_2_).

## 4. Discussion

The purpose of this study was to compare physiological responses, including heart rate, estimated metabolic and pulmonary measures, between two singles pickleball activities, one in MR and one IRL. It was hypothesized that we would observe higher values for heart rate, metabolic and pulmonary measures in real-life gameplay. The data supports our hypothesis, as all the values except FAT% were significantly higher in real-life activity. 

Our findings reveal sufficient evidence to conclude that heart rate measurements obtained were higher during IRL pickleball gameplay. This conclusion aligns with previous literature where in a virtual cycling vs. traditional method the heart rate was lower [5]. However, McClure (2019) [8] investigated whether VR technology has an effect on heart rate among 29 adults. Participants were randomly assigned to one of two 6 min conditions: (i) bicycling at a moderate intensity, (ii) riding in a landscape at moderate intensity while wearing VR. The results revealed that VR coupled with exercise results in an increase in heart rate because participants were able to remove body sensation allowing them to workout longer. In contrast, VR with exercise decreased participants’ HR in a real-life vs. VR condition [5]. The discrepancy between the findings of the present study and those of McClure and Schofield (2020) [8], who reported a higher heart rate in a VR condition compared to a non-VR cycling workout, may be attributed to key differences in activity type and physical constraints. While their study involved stationary cycling, a closed-skill activity with minimal variation in movement intensity, this study examined pickleball, an open-skill sport that requires dynamic, multidirectional movements and reactive gameplay [24]. In the real-world pickleball condition, participants likely engaged in higher-intensity activity due to the greater physical demands and motivating effect of social dynamics, such as competition, cooperation, and peer observation, which are known to enhance effort and engagement during physical activity. Conversely, in our mixed reality pickleball condition, movement was constrained by safety considerations, limited tracking space, and users’ natural hesitance to move freely while wearing a headset. These limitations likely reduced the overall physical workload, resulting in a lower heart rate [25]. Furthermore, while VR cycling may increase arousal through immersive visual stimuli, such effects may not compensate for the reduced physical exertion in a MR simulation of a complex sport like pickleball.

When considering the comparison of pulmonary measures, much less literature exists for real-life vs. virtual reality measures than for heart rate. Our study appears to be the first to report pulmonary measures IRL vs. MR. In a previously published abstract [26], where we compared virtual reality vs. real-life exercising, walking on a VR treadmill seems to increase only the number of breaths taken, while tending to decrease the depth of breath [26]. This comparison showed no difference between VE and VT, while in this experiment, we found differences between all the pulmonary measures, including VT, Rf, and VE. In the wearable-device validity study by Navalta et al. (2024) [27], ventilation (VE) was measured during simulated real-life pickleball gameplay. Compared to our MR condition, their findings also showed higher VE, which aligns with our observation that VE was significantly lower in MR gameplay. In a similar study, it was previously shown that a range of 36–40 ± 5 for Rf and 0.96–1.83 ± 0.22 for VT is close to our Rf and VT data in a real-life setting [27].

As the only comparable data in metabolic measures due to lack of literature, a study by Godfrey compared VO_2_ among four different conditions: VR exergames Beat Saber (BS) and Thrill of the Fight (TOF), one treadmill walking, and one seated video game. Of these conditions, VO_2_ during both VR environments was higher than seated game and lower than during treadmill walking [28]. In some studies, claiming that they are reporting physiological measures, it is hard to directly compare VO_2_ because this variable was not reported by the authors [7,29].

This study contributes to the literature in several important ways. First, it is the first to report pulmonary responses during mixed reality pickleball. Second, it employed direct physiological measurements (e.g., heart rate, VO_2_), which offer objective insights beyond subjective reporting. Third, the comparison of a complex, dynamic sport like pickleball in both real-life and MR conditions provides ecological validity often missing in simpler exergame studies.

The primary real-world application of this research is that the usage of MR can help individuals practice their pickleball or any other athletic skills with less physiological stress, potentially leading to fewer injuries [30]. Since pickleball is increasing in popularity, especially in the United States, there may also be an increase in the number of relative pickleball injuries [16]. It is important to explore modalities that may lower the incidence of injuries among pickleball players, especially those who may be deconditioned. By using MR, individuals may train with less physiological stress and improve skill and conditioning [31], leading to reduced incidence of injury. This research has demonstrated that playing in an MR environment results in a lower heart rate, potentially allowing individuals to practice in a less physiological stressful environment to get ready to meet their exercise goals for this sport IRL. Another implication of this research is that individuals using a VR headset can benefit from energy expended from participating in the activity while enjoying play in a relatively easier MR setting. 

Recent findings further expand the scope of mixed and virtual reality research by showing that physiological and immune responses can be triggered even in the absence of physical exertion or direct pathogen exposure. For instance, studies of VR-based exergames, such as virtual boxing, demonstrate that immersive environments can elicit metabolic and cardiovascular responses comparable to traditional exercise modalities, underscoring the potential of VR to promote health benefits through engaging gameplay [32]. Beyond physical exertion, evidence now suggests that virtual stimuli alone may modulate biological systems. Trabanelli et al. (2025) [33] reported that exposure to avatars displaying signs of infection within peripersonal space in VR activated neural circuits associated with threat detection and, remarkably, initiated an anticipatory immune response involving innate lymphoid cells, comparable to responses observed after real infection challenges to the immune system. Together, these studies highlight that virtual and mixed reality experiences extend their impact beyond perceptual and behavioral domains to physiological and even immunological processes. This reinforces the notion that while MR pickleball may produce attenuated cardiovascular and pulmonary responses compared to real-life play, the immersive qualities of MR can still provoke meaningful systemic adaptations, suggesting opportunities for both safe training and novel therapeutic applications.

Several limitations should be considered when interpreting the results of this study. First, a warm-up was performed prior to MR testing which could have an effect on physiological responses via raising the heart rate. Second, pickleball might not be a steady-state activity most of the time, which can influence VO_2_ measurements. To account for this, VO_2_ values were averaged over the final two minutes of gameplay. Another limitation is that participants’ prior experience with virtual reality or mixed reality environments was not assessed. This factor may have influenced how naturally they interacted with the MR condition, for example, by limiting physical engagement or longer reaction time. Participants’ unfamiliarity with pickleball may have affected their performance and physiological responses, for example, by lower physical engagement or less movement efficiency. Additionally, differences in paddle and controller weight between the MR and IRL conditions may have influenced gameplay mechanics and energy expenditure. A limitation may be the relatively small sample size tested in the current investigation; however, medium to large effect sizes suggest an adequate number were tested to answer the questions posed in the present study. Future research should consider measuring MR familiarity, and the design and use of a standardized questionnaire to better understand the impact of prior exposure on physiological outcomes. Future studies should also consider exploring the long-term effects of MR training on skill acquisition and injury prevention. Additionally, incorporating motion tracking and biomechanical analysis may provide further insight into movement quality and the physical demands of gameplay across environments. Future studies should also account for paddle and controller weight to ensure that equipment differences do not confound physiological or performance outcomes. Including more diverse populations (e.g., older adults or experienced athletes) may also help clarify whether MR can be adapted for a broader range of users. Future studies could also examine potential sex differences to determine whether physiological and performance responses vary by gender.

## 5. Conclusions

In conclusion, this study provides novel evidence that key physiological measures including HR, Rf, VT, VE, RER, CHO%, EE, and VO_2_ are consistently lower during MR pickleball compared to real-life play. These findings suggest that MR may offer a lower-intensity alternative to traditional gameplay. Given this reduced physiological load, future research should investigate whether MR-based pickleball can lower injury risk, particularly among deconditioned or older adults. MR training may also serve as a steppingstone for novice participants, enabling gradual adaptation to the physical and cognitive demands of real-world pickleball. Additionally, its controlled, immersive nature could make MR a valuable tool in rehabilitation settings, where minimizing physical strain is critical while still encouraging engagement and movement.

## Data Availability

The data presented in this study are available on request from the corresponding author.

## Abbreviations

The following abbreviations are used in this manuscript:

MR	Mixed reality
IRL	In real life
HR	Heart rate
Rf	Respiratory frequency
VT	Tidal volume
VO_2_	Volume of oxygen
VE	Ventilation
CHO%	Percentage of calories from carbohydrate
FAT%	Percentage of calories from fat
EE	Energy expenditure
RER	Respiratory exchange ratio
